# The genome sequence of the moss carder bee, *Bombus muscorum *(Linnaeus, 1758)

**DOI:** 10.12688/wellcomeopenres.22739.1

**Published:** 2024-07-22

**Authors:** Gavin R. Broad, Ian Barnes, Natural History Museum Genome Acquisition Lab, Darwin Tree of Life Barcoding collective, Wellcome Sanger Institute Tree of Life Management, Samples and Laboratory team, Wellcome Sanger Institute Scientific Operations: Sequencing Operations, Wellcome Sanger Institute Tree of Life Core Informatics team, Tree of Life Core Informatics collective, Darwin Tree of Life Consortium

**Affiliations:** 1 Natural History Museum, London, England, UK

**Keywords:** Bombus muscorum, moss carder bee, genome sequence, chromosomal, Hymenoptera

## Abstract

We present a genome assembly from an individual female *Bombus muscorum* (the moss carder bee; Arthropoda; Insecta; Hymenoptera; Apidae). The genome sequence spans 317.70 megabases. Most of the assembly is scaffolded into 17 chromosomal pseudomolecules. The mitochondrial genome has also been assembled and is 21.15 kilobases in length. Gene annotation of this assembly on Ensembl identified 11,668 protein-coding genes.

## Species taxonomy

Eukaryota; Opisthokonta; Metazoa; Eumetazoa; Bilateria; Protostomia; Ecdysozoa; Panarthropoda; Arthropoda; Mandibulata; Pancrustacea; Hexapoda; Insecta; Dicondylia; Pterygota; Neoptera; Endopterygota; Hymenoptera; Apocrita; Aculeata; Apoidea; Anthophila; Apidae; Apinae; Bombini; Bombus; *Bombus*; *Bombus muscorum* (Linnaeus, 1758) (NCBI:txid203813).

## Background

The moss carder bee, *Bombus muscorum*, is a bumblebee species that is sparsely but widely distributed across Europe and parts of Asia. It is primarily found in habitats characterised by open, flower-rich grasslands, such as meadows and heathland. Within the geographic range of the Darwin Tree of Life project, its preference for damper habitats means that it is typically found in bogs, marshes, moorlands, and coastal grasslands like machair. Formerly widespread, it is now found only in small, fragmented populations, although it is still abundant on some Scottish islands ( Edwards & Broad, 2005). The species is well adapted to these environments as it employs mosses for nest construction, a behaviour known as “carding”. Nests are constructed on the surface of the ground, typically in grass fields, moss, and marshy heath. Fewer workers are produced than other bumblebee species, with the total number of workers in British colonies estimated at fewer than 100, and often much fewer than 100 ( Benton, 2006).

*Bombus muscorum* is one of three all-brown bumblebee species in Britain and Ireland, along with *B. pascuorum* and *B. humilis*. While it is relatively easy to separate these species from other members of genus *Bombus*, differentiating between these three can be difficult. *B. muscorum* has no black hairs on the dorsal surface of the abdomen (unlike *B. pascuorum*), and also has no black hairs on the dorsum of the thorax. *Bombus humilis* has a thin scattering of black hairs around and above the wing bases.

The moss carder bee is an annual species with a seasonal life cycle. The queens emerge from hibernation in late spring, typically in May, with workers present from June onwards. It is polylectic, visiting a wide variety of plant species with a preference for flowers in the families Fabaceae, Scrophulariaceae, Orobanchaceae, Lamiaceae, and Asteraceae, including clover, deadnettle, yellow rattle and knapweed. This is a “long-tongued” bumblebee, and thus a pollinator of plants with long corollas ( Edwards & Jenner, 2005).

The limited dispersal capabilities of this species may be a component in its decline, and previous genetic work has suggested that surviving populations of this rare insect suffer from inbreeding caused by geographical isolation ( Darvill *et al.*, 2006; Darvill *et al.*, 2010). This chromosomally complete genome sequence for *B. muscorum*, based on one female specimen from Haroldswick, Scotland, could therefore facilitate studies into the conservation of this declining pollinator, and particularly the spatial distribution of different populations and the extent of gene flow and inbreeding.

## Genome sequence report

**Figure 1.**
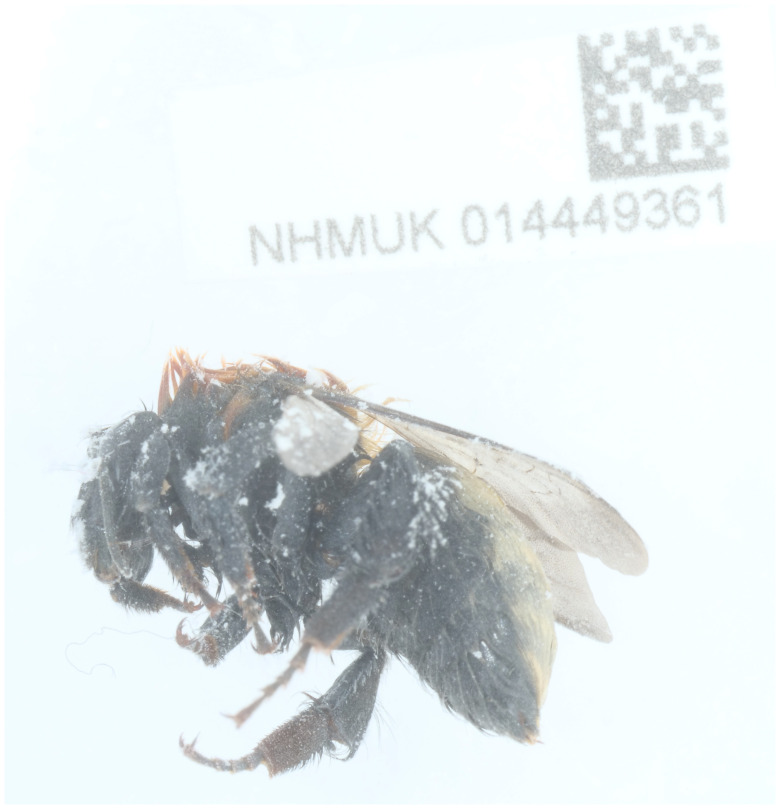
Photograph of the *Bombus muscorum* (iyBomMusc1) specimen used for genome sequencing.

The genome of an adult female *Bombus muscorum* ( Figure 1) was sequenced using Pacific Biosciences single-molecule HiFi long reads, generating a total of 29.34 Gb (gigabases) from 2.44 million reads, providing approximately 82-fold coverage. Primary assembly contigs were scaffolded with chromosome conformation Hi-C data, which produced 127.43 Gbp from 843.94 million reads, yielding an approximate coverage of 401-fold. Information about the sample and sequencing is summarised in Table 1.

**Table 1.** Specimen and sequencing data for *Bombus muscorum*.

Project information			
**Study title**	Bombus muscorum		
**Umbrella BioProject**	PRJEB59139		
**Species**	*Bombus muscorum*		
**BioSample**	SAMEA14448357		
**NCBI taxonomy ID**	203813		
Specimen information			
Technology	ToLID	BioSample accession	Organism part
**PacBio long read sequencing**	iyBomMusc1	SAMEA14448603	thorax
**Hi-C sequencing**	iyBomMusc1	SAMEA14448602	head
**RNA sequencing**	iyBomMusc1	SAMEA14448603	thorax
Sequencing information			
Platform	Run accession	Read count	Base count (Gb)
**Hi-C Illumina NovaSeq 6000**	ERR10802469	8.44e+08	127.43
**PacBio Sequel IIe**	ERR10812842	2.44e+06	29.34
**RNA Illumina NovaSeq 6000**	ERR11837462	7.25e+07	10.95

**Figure 2.**
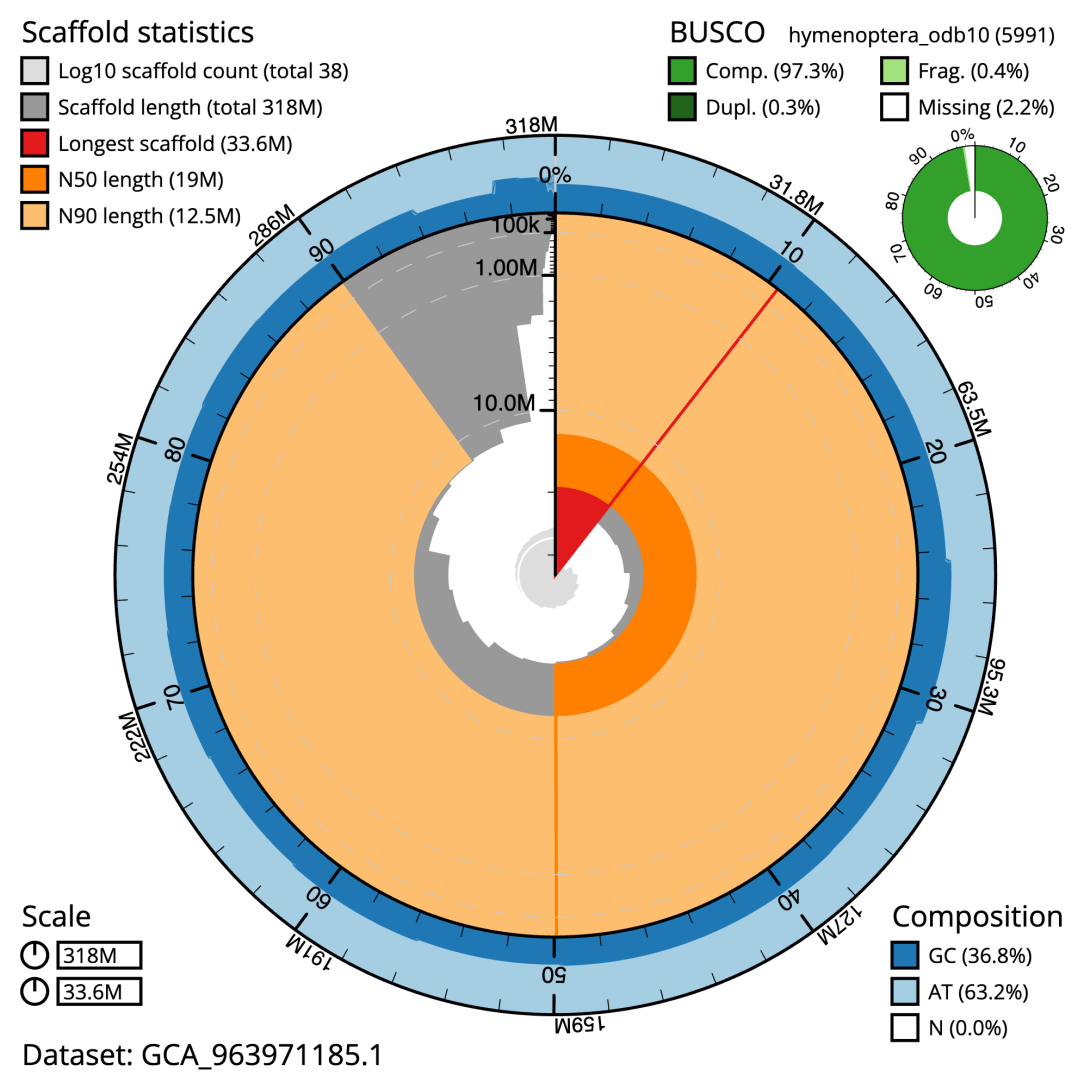
Genome assembly of *Bombus muscorum*, iyBomMusc1.1: metrics. The BlobToolKit snail plot shows N50 metrics and BUSCO gene completeness. The main plot is divided into 1,000 size-ordered bins around the circumference with each bin representing 0.1% of the 317,697,559 bp assembly. The distribution of scaffold lengths is shown in dark grey with the plot radius scaled to the longest scaffold present in the assembly (33,605,962 bp, shown in red). Orange and pale-orange arcs show the N50 and N90 scaffold lengths (19,019,874 and 12,512,728 bp), respectively. The pale grey spiral shows the cumulative scaffold count on a log scale with white scale lines showing successive orders of magnitude. The blue and pale-blue area around the outside of the plot shows the distribution of GC, AT and N percentages in the same bins as the inner plot. A summary of complete, fragmented, duplicated and missing BUSCO genes in the hymenoptera_odb10 set is shown in the top right. An interactive version of this figure is available at https://blobtoolkit.genomehubs.org/view/Bombus_muscorum/dataset/GCA_963971185.1/snail.

**Figure 3.**
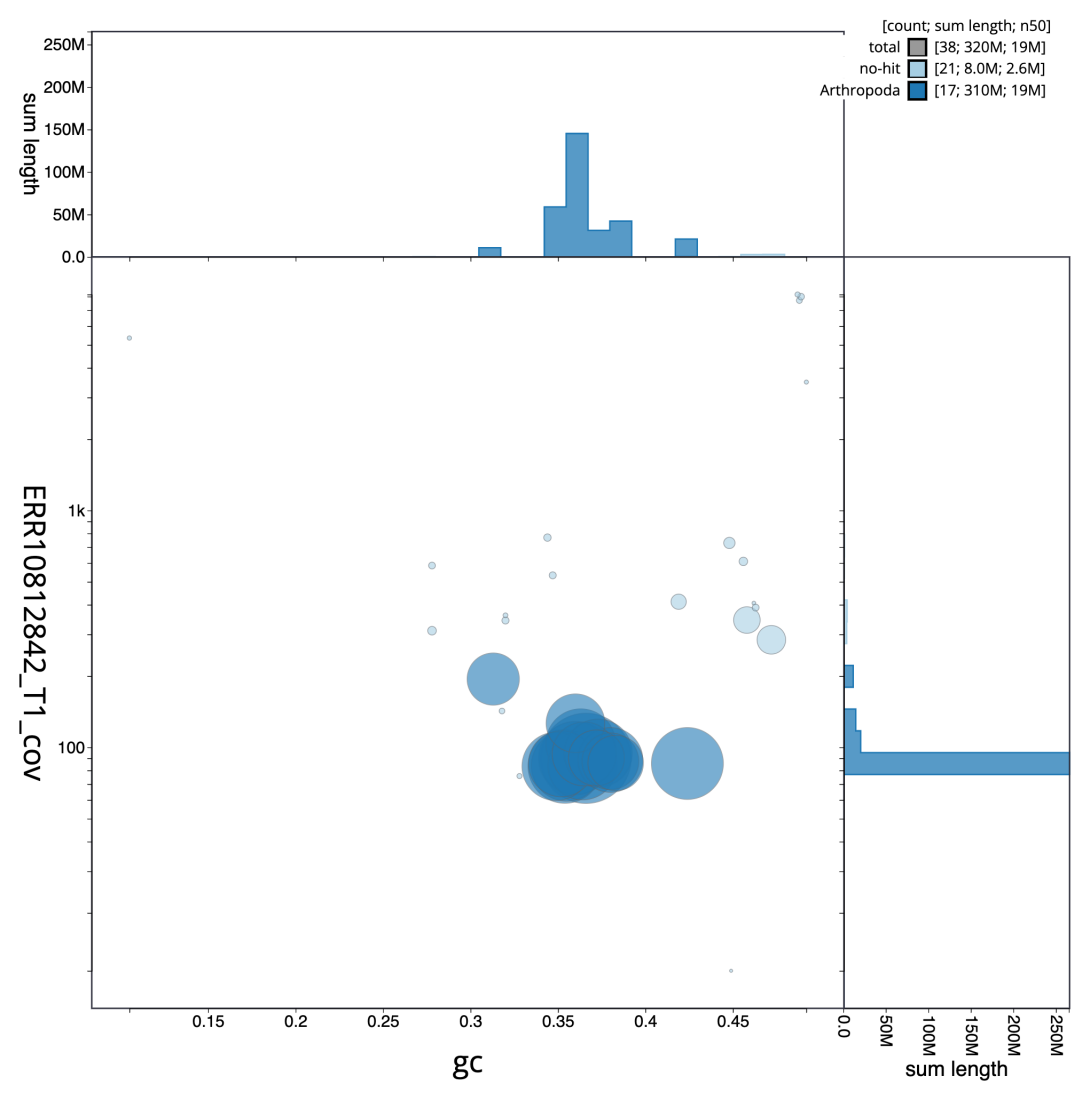
Genome assembly of *Bombus muscorum*, iyBomMusc1.1: BlobToolKit GC-coverage plot. Sequences are coloured by phylum. Circles are sized in proportion to sequence length. Histograms show the distribution of sequence length sum along each axis. An interactive version of this figure is available at https://blobtoolkit.genomehubs.org/view/Bombus_muscorum/dataset/GCA_963971185.1/blob.

**Figure 4.**
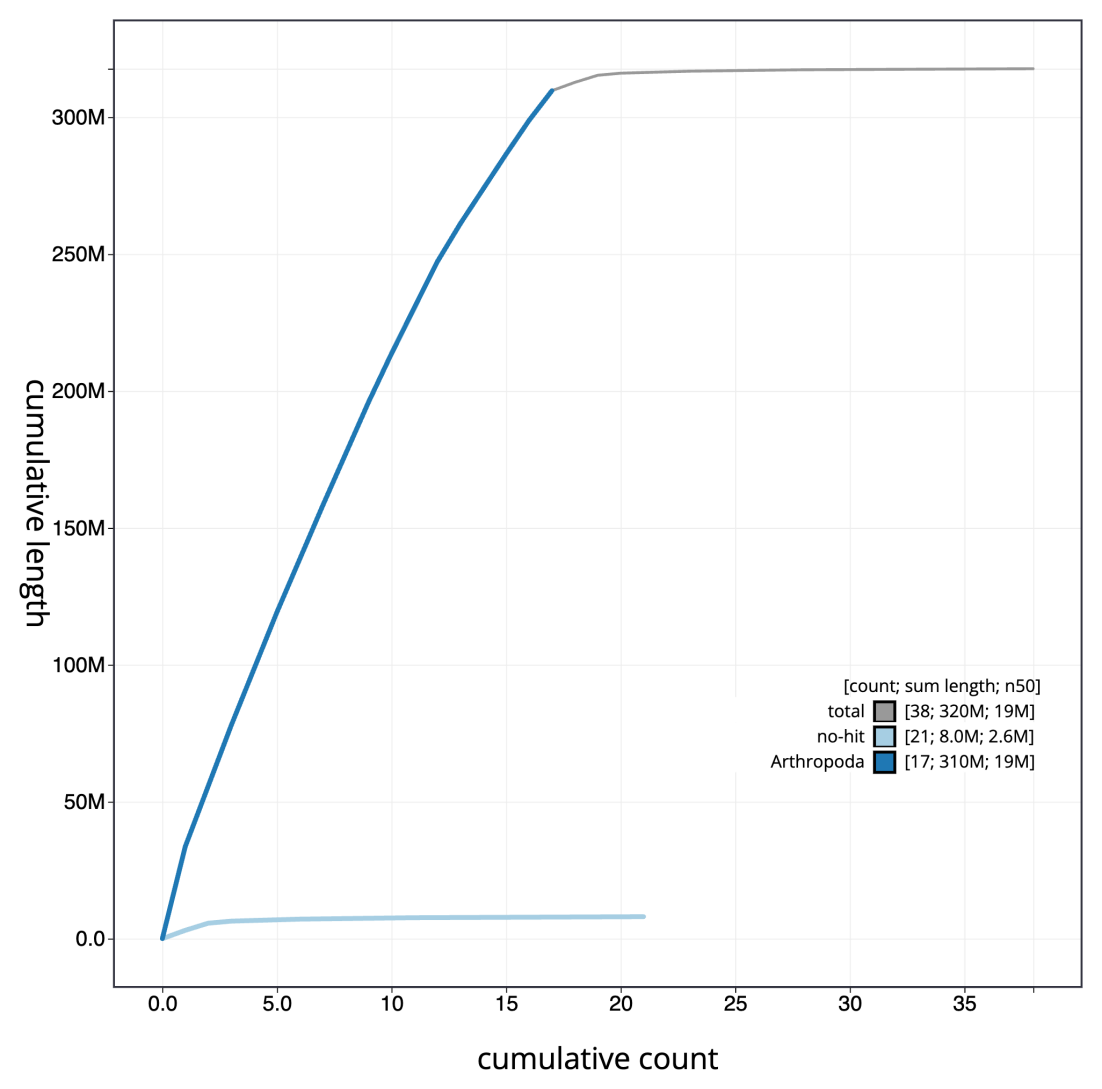
Genome assembly of *Bombus muscorum* iyBomMusc1.1: BlobToolKit cumulative sequence plot. The grey line shows cumulative length for all sequences. Coloured lines show cumulative lengths of sequences assigned to each phylum using the buscogenes taxrule. An interactive version of this figure is available at https://blobtoolkit.genomehubs.org/view/Bombus_muscorum/dataset/GCA_963971185.1/cumulative.

**Figure 5.**
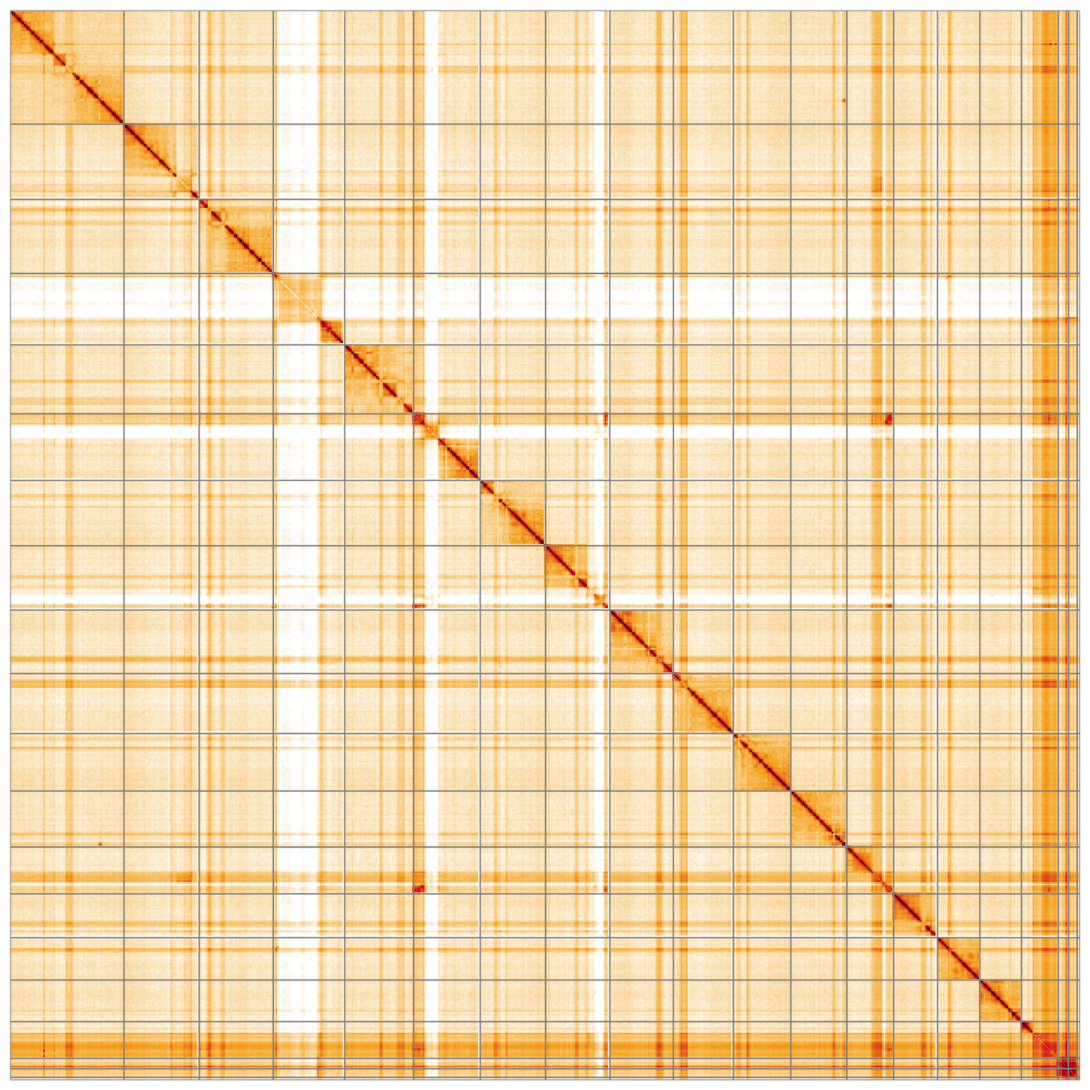
Genome assembly of *Bombus muscorum* iyBomMusc1.1: Hi-C contact map of the iyBomMusc1.1 assembly, visualised using HiGlass. Chromosomes are shown in order of size from left to right and top to bottom. An interactive version of this figure may be viewed at https://genome-note-higlass.tol.sanger.ac.uk/l/?d=Ky3BTPf5R2KbDM-OqUDefw.

Manual assembly curation corrected 55 missing joins or mis-joins and 3 haplotypic duplications, reducing the assembly length by 0.45% and the scaffold number by 30.91%, and increasing the scaffold N50 by 14.79%. The final assembly has a total length of 317.70 Mb in 37 sequence scaffolds with a scaffold N50 of 19.0 Mb ( Table 2). The total count of gaps in the scaffolds is 126. The snail plot in Figure 2 provides a summary of the assembly statistics, while the distribution of assembly scaffolds on GC proportion and coverage is shown in Figure 3. The cumulative assembly plot in Figure 4 shows curves for subsets of scaffolds assigned to different phyla. Most (97.47%) of the assembly sequence was assigned to 17 chromosomal-level scaffolds. Chromosome-scale scaffolds confirmed by the Hi-C data are named in order of size ( Figure 5; Table 3). While not fully phased, the assembly deposited is of one haplotype. Contigs corresponding to the second haplotype have also been deposited. The mitochondrial genome was also assembled and can be found as a contig within the multifasta file of the genome submission.

**Table 2.** Genome assembly data for *Bombus muscorum*, iyBomMusc1.1.

Genome assembly		
Assembly name	iyBomMusc1.1	
Assembly accession	GCA_963971185.1	
*Accession of alternate haplotype*	*GCA_963971125.1*	
Span (Mb)	317.70	
Number of contigs	164	
Contig N50 length (Mb)	3.4	
Number of scaffolds	37	
Scaffold N50 length (Mb)	19.0	
Longest scaffold (Mb)	33.61	
Assembly metrics *		*Benchmark*
Consensus quality (QV)	62.7	*≥ 50*
*k*-mer completeness	100.0%	*≥ 95%*
BUSCO **	C:97.2%[S:96.9%,D:0.3%],F:0.5%,M:2.3%,n:5,991	*C ≥ 95%*
Percentage of assembly mapped to chromosomes	97.47%	*≥ 95%*
Sex chromosomes	None	*localised homologous pairs*
Organelles	Mitochondrial genome: 21.15 kb	*complete single alleles*
Genome annotation of assembly GCA_963971185.1 at Ensembl		
Number of protein-coding genes	11,668	
Number of non-coding genes	4,724	
Number of gene transcripts	30,785	

* Assembly metric benchmarks are adapted from column VGP-2020 of “Table 1: Proposed standards and metrics for defining genome assembly quality” from Rhie *et al.* (2021). ** BUSCO scores based on the hymenoptera_odb10 BUSCO set using version 5.4.3. C = complete [S = single copy, D = duplicated], F = fragmented, M = missing, n = number of orthologues in comparison. A full set of BUSCO scores is available at https://blobtoolkit.genomehubs.org/view/Bombus_muscorum/dataset/GCA_963971185.1/busco.

**Table 3.** Chromosomal pseudomolecules in the genome assembly of *Bombus muscorum*, iyBomMusc1.

INSDC accession	Name	Length (Mb)	GC%
OZ020127.1	1	33.61	36.5
OZ020128.1	2	22.21	35.5
OZ020129.1	3	21.93	36.0
OZ020130.1	4	21.08	42.5
OZ020131.1	5	20.39	35.5
OZ020132.1	6	19.66	36.5
OZ020133.1	7	19.31	36.5
OZ020134.1	8	19.02	35.0
OZ020135.1	9	18.79	37.5
OZ020136.1	10	17.69	35.0
OZ020137.1	11	16.98	38.0
OZ020138.1	12	16.57	36.5
OZ020139.1	13	13.86	36.0
OZ020140.1	14	12.92	38.0
OZ020141.1	15	12.51	37.0
OZ020142.1	16	12.3	38.5
OZ020143.1	17	10.87	31.5
OZ020144.1	MT	0.02	10.5

The estimated Quality Value (QV) of the final assembly is 62.7 with *k*-mer completeness of 100.0%, and the assembly has a BUSCO v5.4.3 completeness of 97.2% (single = 96.9%, duplicated = 0.3%), using the hymenoptera_odb10 reference set ( *n* = 5,991).

Metadata for specimens, BOLD barcode results, spectra estimates, sequencing runs, contaminants and pre-curation assembly statistics are given at https://links.tol.sanger.ac.uk/species/203813.

## Genome annotation report

The *Bombus muscorum* genome assembly (GCA_963971185.1) was annotated at the European Bioinformatics Institute (EBI) on Ensembl Rapid Release. The resulting annotation includes 30,785 transcribed mRNAs from 11,668 protein-coding and 4,724 non-coding genes ( Table 2; https://rapid.ensembl.org/Bombus_muscorum_GCA_963971185.1/Info/Index). The average transcript length is 12,284.80. There are 1.88 coding transcripts per gene and 6.23 exons per transcript.

## Methods

### Sample acquisition

An adult female *Bombus muscorum* (specimen ID NHMUK014449361, ToLID iyBomMusc1) was collected from Haroldswick, Scotland, UK (latitude 60.79, longitude –0.83) on 2021-07-30 by sweeping. The specimen was collected and identified by Gavin Broad (Natural History Museum) and preserved by dry freezing at – 80°C.

In addition to identification based on morphology, the species taxonomy was verified by DNA barcoding soon after collection, according to the framework developed by Twyford *et al*. (2024). A small sample was dissected from the specimen and stored in ethanol. The tissue was lysed, and the COI marker region was amplified by PCR. Amplicons were sequenced and compared to the BOLD database, confirming the species identification ( Crowley *et al.*, 2023). The standard operating procedures for the Darwin Tree of Life barcoding have been deposited on protocols.io ( Beasley *et al.*, 2023). The remaining parts of the specimen were shipped on dry ice to the Wellcome Sanger Institute (WSI). A DNA barcode was also generated from the PacBio sequencing data at a later stage for sample tracking through the genome production pipeline at the WSI ( Twyford *et al.*, 2024).

### Nucleic acid extraction

The workflow for high molecular weight (HMW) DNA extraction at the WSI Tree of Life Core Laboratory includes a sequence of core procedures: sample preparation; sample homogenisation, DNA extraction, fragmentation, and clean-up. In sample preparation, the iyBomMusc1 sample was weighed and dissected on dry ice ( Jay *et al.*, 2023). Tissue from thorax was homogenised using a PowerMasher II tissue disruptor ( Denton *et al.*, 2023a).

HMW DNA was extracted using the Automated MagAttract v1 protocol ( Sheerin *et al.*, 2023). DNA was sheared into an average fragment size of 12–20 kb in a Megaruptor 3 system ( Todorovic *et al.*, 2023). Sheared DNA was purified by solid-phase reversible immobilisation ( Strickland *et al.*, 2023): in brief, the method employs a 1.8X ratio of AMPure PB beads to sample to eliminate shorter fragments and concentrate the DNA. The concentration of the sheared and purified DNA was assessed using a Nanodrop spectrophotometer and Qubit Fluorometer using the Qubit dsDNA High Sensitivity Assay kit. Fragment size distribution was evaluated by running the sample on the FemtoPulse system.

RNA was extracted from thorax tissue of iyBomMusc1 in the Tree of Life Laboratory at the WSI using the RNA Extraction: Automated MagMax™ *mir*Vana protocol ( do Amaral *et al.*, 2023). The RNA concentration was assessed using a Nanodrop spectrophotometer and a Qubit Fluorometer using the Qubit RNA Broad-Range Assay kit. Analysis of the integrity of the RNA was done using the Agilent RNA 6000 Pico Kit and Eukaryotic Total RNA assay.

Protocols developed by the WSI Tree of Life laboratory are publicly available on protocols.io ( Denton *et al.*, 2023b).

### Sequencing

Pacific Biosciences HiFi circular consensus DNA sequencing libraries were constructed according to the manufacturers’ instructions. Poly(A) RNA-Seq libraries were constructed using the NEB Ultra II RNA Library Prep kit. DNA and RNA sequencing was performed by the Scientific Operations core at the WSI on Pacific Biosciences Sequel IIe (HiFi) and Illumina NovaSeq 6000 (RNA-Seq) instruments. Hi-C data were also generated from head tissue of iyBomMusc1 using the Arima-HiC v2 kit. The Hi-C sequencing was performed using paired-end sequencing with a read length of 150 bp on the Illumina NovaSeq 6000 instrument.

### Genome assembly, curation and evaluation

***Assembly*.** The original assembly of HiFi reads was performed using Hifiasm ( Cheng *et al.*, 2021) with the --primary option. Haplotypic duplications were identified and removed with purge_dups ( Guan *et al.*, 2020). Hi-C reads are further mapped with bwa-mem2 ( Vasimuddin *et al.*, 2019) to the primary contigs, which are further scaffolded using the provided Hi-C data ( Rao *et al.*, 2014) in YaHS ( Zhou *et al.*, 2023) using the --break option. Scaffolded assemblies are evaluated using Gfastats ( Formenti *et al.*, 2022), BUSCO ( Manni *et al.*, 2021) and MERQURY.FK ( Rhie *et al.*, 2020).

The mitochondrial genome was assembled using MitoHiFi ( Uliano-Silva *et al.*, 2023), which runs MitoFinder ( Allio *et al.*, 2020) or MITOS ( Bernt *et al.*, 2013) and uses these annotations to select the final mitochondrial contig and to ensure the general quality of the sequence.

***Assembly curation*.** The assembly was decontaminated using the Assembly Screen for Cobionts and Contaminants (ASCC) pipeline (article in preparation). Flat files and maps used in curation were generated in TreeVal ( Pointon *et al.*, 2023). Manual curation was primarily conducted using PretextView ( Harry, 2022), with additional insights provided by JBrowse2 ( Diesh *et al.*, 2023) and HiGlass ( Kerpedjiev *et al.*, 2018). Scaffolds were visually inspected and corrected as described by Howe *et al*. (2021). Any identified contamination, missed joins, and mis-joins were corrected, and duplicate sequences were tagged and removed. The entire process is documented at https://gitlab.com/wtsi-grit/rapid-curation (article in preparation).

***Evaluation of the final assembly*.** The final assembly was post-processed and evaluated with the three Nextflow ( Di Tommaso *et al.*, 2017) DSL2 pipelines “sanger-tol/readmapping” ( Surana *et al.*, 2023a), “sanger-tol/genomenote” ( Surana *et al.*, 2023b), and “sanger-tol/blobtoolkit” ( Muffato *et al.*, 2024). The pipeline sanger-tol/readmapping aligns the Hi-C reads with bwa-mem2 ( Vasimuddin *et al.*, 2019) and combines the alignment files with SAMtools ( Danecek *et al.*, 2021). The sanger-tol/genomenote pipeline transforms the Hi-C alignments into a contact map with BEDTools ( Quinlan & Hall, 2010) and the Cooler tool suite ( Abdennur & Mirny, 2020), which is then visualised with HiGlass ( Kerpedjiev *et al.*, 2018). It also provides statistics about the assembly with the NCBI datasets ( Sayers *et al.*, 2024) report, computes *k*-mer completeness and QV consensus quality values with FastK and MERQURY.FK, and a completeness assessment with BUSCO ( Manni *et al.*, 2021).

The sanger-tol/blobtoolkit pipeline is a Nextflow port of the previous Snakemake Blobtoolkit pipeline ( Challis *et al.*, 2020). It aligns the PacBio reads with SAMtools and minimap2 ( Li, 2018) and generates coverage tracks for regions of fixed size. In parallel, it queries the GoaT database ( Challis *et al.*, 2023) to identify all matching BUSCO lineages to run BUSCO ( Manni *et al.*, 2021). For the three domain-level BUSCO lineage, the pipeline aligns the BUSCO genes to the Uniprot Reference Proteomes database ( Bateman *et al.*, 2023) with DIAMOND ( Buchfink *et al.*, 2021) blastp. The genome is also split into chunks according to the density of the BUSCO genes from the closest taxonomically lineage, and each chunk is aligned to the Uniprot Reference Proteomes database with DIAMOND blastx. Genome sequences that have no hit are then chunked with seqtk and aligned to the NT database with blastn ( Altschul *et al.*, 1990). All those outputs are combined with the blobtools suite into a blobdir for visualisation.

The evaluation pipelines were developed using the nf-core tooling ( Ewels *et al.*, 2020), use MultiQC ( Ewels *et al.*, 2016), and make extensive use of the Conda package manager, the Bioconda initiative ( Grüning *et al.*, 2018), the Biocontainers infrastructure ( da Veiga Leprevost *et al.*, 2017), and the Docker ( Merkel, 2014) and Singularity ( Kurtzer *et al.*, 2017) containerisation solutions.

Table 4 contains a list of relevant software tool versions and sources.

**Table 4.** Software tools: versions and sources.

Software tool	Version	Source
BEDTools	2.30.0	https://github.com/arq5x/bedtools2
BLAST	2.14.0	ftp://ftp.ncbi.nlm.nih.gov/blast/executables/blast+/
BlobToolKit	4.3.7	https://github.com/blobtoolkit/blobtoolkit
BUSCO	5.4.3 and 5.5.0	https://gitlab.com/ezlab/busco
bwa-mem2	2.2.1	https://github.com/bwa-mem2/bwa-mem2
Cooler	0.8.11	https://github.com/open2c/cooler
DIAMOND	2.1.8	https://github.com/bbuchfink/diamond
fasta_windows	0.2.4	https://github.com/tolkit/fasta_windows
FastK	427104ea91c78c3b8b8b49f1a7d6bbeaa869ba1c	https://github.com/thegenemyers/FASTK
Gfastats	1.3.6	https://github.com/vgl-hub/gfastats
GoaT CLI	0.2.5	https://github.com/genomehubs/goat-cli
Hifiasm	0.16.1-r375	https://github.com/chhylp123/hifiasm
HiGlass	44086069ee7d4d3f6f3f0012569789ec138f42b84aa44357826c0b6753eb28de	https://github.com/higlass/higlass
Merqury.FK	d00d98157618f4e8d1a9190026b19b471055b22e	https://github.com/thegenemyers/MERQURY.FK
MitoHiFi	2	https://github.com/marcelauliano/MitoHiFi
MultiQC	1.14, 1.17, and 1.18	https://github.com/MultiQC/MultiQC
NCBI Datasets	15.12.0	https://github.com/ncbi/datasets
Nextflow	23.04.0-5857	https://github.com/nextflow-io/nextflow
PretextView	0.2	https://github.com/sanger-tol/PretextView
purge_dups	1.2.3	https://github.com/dfguan/purge_dups
samtools	1.16.1, 1.17, and 1.18	https://github.com/samtools/samtools
sanger-tol/ascc	-	https://github.com/sanger-tol/ascc
sanger-tol/genomenote	1.1.1	https://github.com/sanger-tol/genomenote
sanger-tol/readmapping	1.2.1	https://github.com/sanger-tol/readmapping
Seqtk	1.3	https://github.com/lh3/seqtk
Singularity	3.9.0	https://github.com/sylabs/singularity
TreeVal	1.0.0	https://github.com/sanger-tol/treeval
YaHS	1.2a	https://github.com/c-zhou/yahs

### Genome annotation

The Ensembl Genebuild annotation system ( Aken *et al.*, 2016) was used to generate annotation for the *Bombus muscorum* assembly (GCA_963971185.1) in Ensembl Rapid Release at the EBI. Annotation was created primarily through alignment of transcriptomic data to the genome, with gap filling via protein-to-genome alignments of a select set of proteins from UniProt ( UniProt Consortium, 2019).

### Wellcome Sanger Institute – Legal and Governance

The materials that have contributed to this genome note have been supplied by a Darwin Tree of Life Partner. The submission of materials by a Darwin Tree of Life Partner is subject to the **‘Darwin Tree of Life Project Sampling Code of Practice’**, which can be found in full on the Darwin Tree of Life website here. By agreeing with and signing up to the Sampling Code of Practice, the Darwin Tree of Life Partner agrees they will meet the legal and ethical requirements and standards set out within this document in respect of all samples acquired for, and supplied to, the Darwin Tree of Life Project.

Further, the Wellcome Sanger Institute employs a process whereby due diligence is carried out proportionate to the nature of the materials themselves, and the circumstances under which they have been/are to be collected and provided for use. The purpose of this is to address and mitigate any potential legal and/or ethical implications of receipt and use of the materials as part of the research project, and to ensure that in doing so we align with best practice wherever possible. The overarching areas of consideration are:

• Ethical review of provenance and sourcing of the material

• Legality of collection, transfer and use (national and international)

Each transfer of samples is further undertaken according to a Research Collaboration Agreement or Material Transfer Agreement entered into by the Darwin Tree of Life Partner, Genome Research Limited (operating as the Wellcome Sanger Institute), and in some circumstances other Darwin Tree of Life collaborators.

## Data Availability

European Nucleotide Archive: *Bombus muscorum*. Accession number PRJEB59139; https://identifiers.org/ena.embl/PRJEB59139 ( Wellcome Sanger Institute, 2024). The genome sequence is released openly for reuse. The *Bombus muscorum* genome sequencing initiative is part of the Darwin Tree of Life (DToL) project. All raw sequence data and the assembly have been deposited in INSDC databases. The genome will be annotated using available RNA-Seq data and presented through the Ensembl pipeline at the European Bioinformatics Institute. Raw data and assembly accession identifiers are reported in Table 1 and Table 2.

## Author information

Members of the Natural History Museum Genome Acquisition Lab are listed here: https://doi.org/10.5281/zenodo.7139035.

Members of the Darwin Tree of Life Barcoding collective are listed here: https://doi.org/10.5281/zenodo.4893703.

Members of the Wellcome Sanger Institute Tree of Life Management, Samples and Laboratory team are listed here: https://doi.org/10.5281/zenodo.10066175.

Members of Wellcome Sanger Institute Scientific Operations: Sequencing Operations are listed here: https://doi.org/10.5281/zenodo.10043364.

Members of the Wellcome Sanger Institute Tree of Life Core Informatics team are listed here: https://doi.org/10.5281/zenodo.10066637.

Members of the Tree of Life Core Informatics collective are listed here: https://doi.org/10.5281/zenodo.5013541.

Members of the Darwin Tree of Life Consortium are listed here: https://doi.org/10.5281/zenodo.4783558.
