# Supplementary material for: The genome sequence of the moss carder bee, Bombus muscorum (Linnaeus, 1758)
Source: Wellcome Open Res. 2024 Jul 22;9:397. [Version 1] doi: 10.12688/wellcomeopenres.22739.1 (PMC11484540; doi:10.12688/wellcomeopenres.22739.1)
Supplement: Peer review history

# Reviewer report: Reviewer response for version 1

**DOI:** 10.21956/wellcomeopenres.25042.r100984

**Published:** 2024-10-16

**Authors:** Leonardo Tresoldi Gonçalves

**Affiliations:** 1 Universidade Federal do Rio Grande do Sul, Porto Alegre, State of Rio Grande do Sul, Brazil

The paper presents a high-quality reference genome for the moss carder bee, *Bombus muscorum*. The paper is well-written and the methodology for obtaining the dataset is clearly explained. 

**I only have the following suggestions:**

 1. Please revise the Background section. Several of the sentences about the biology of *B. muscorum* lack references.

 2. The authors mentioned they used MitoHiFi to assemble the mitochondrial genome, a program that "runs MitoFinder or MITOS and uses these annotations (..)". However, it remains unclear which mode of MitoHiFi was employed for annotation – MitoFinder or MITOS. Please specify.

Are sufficient details of methods and materials provided to allow replication by others?

Yes

Is the rationale for creating the dataset(s) clearly described?

Yes

Are the datasets clearly presented in a useable and accessible format?

Yes

Are the protocols appropriate and is the work technically sound?

Yes

Reviewer Expertise:

Phylogenetics, phylogenomics, genomics, DNA barcoding.

I confirm that I have read this submission and believe that I have an appropriate level of expertise to confirm that it is of an acceptable scientific standard.

# Reviewer report: Reviewer response for version 1

**DOI:** 10.21956/wellcomeopenres.25042.r102868

**Published:** 2024-10-04

**Authors:** Jiangli Tan

**Affiliations:** 1 Northwest University, Xi An, China

The article is well-prepared, contains valuable data, and follows appropriate scientific standards. It is suitable for publication, especially for a specialized audience interested in genomic research, biodiversity, and conservation.

**Suggestion:**

- While the article mentions that the genome can aid conservation, briefly discuss how this data could practically impact conservation strategies (e.g., genetic diversity analysis, habitat restoration) would strengthen the article's contribution.

Are sufficient details of methods and materials provided to allow replication by others?

Yes

Is the rationale for creating the dataset(s) clearly described?

Yes

Are the datasets clearly presented in a useable and accessible format?

Yes

Are the protocols appropriate and is the work technically sound?

Yes

Reviewer Expertise:

taxonomy on Hymenoptera

I confirm that I have read this submission and believe that I have an appropriate level of expertise to confirm that it is of an acceptable scientific standard.
